# A Structured Myofascial Pain Management Program for Chronic Headache Including Medication-Refractory Cases

**DOI:** 10.7759/cureus.91095

**Published:** 2025-08-27

**Authors:** Sho Usuda, Satoru Morikawa, Hironori Saisu, Takazumi Yasui, Tsubasa Takizawa, Taneaki Nakagawa, Wataru Muraoka

**Affiliations:** 1 Department of Dentistry and Oral Surgery, Keio University School of Medicine, Tokyo, JPN; 2 Multidisciplinary Pain Center, Aichi Medical University, Nagakute, JPN; 3 Department of Neurology, Keio University School of Medicine, Tokyo, JPN

**Keywords:** chronic headache, ipos, medication-refractory headache, muscle palpation, myofascial pain, nonpharmacological intervention, pain management, self-care, temporomandibular disorder

## Abstract

Background and objective

Musculoskeletal factors, such as myofascial pain, are often overlooked in chronic headaches. This study aimed to evaluate the effectiveness of a structured self-care program, myofascial pain management (MPM), for patients with chronic headaches.

Methods

This single-arm observational study involved 37 patients with chronic headaches who were referred from a neurology clinic. The patients were categorized into two groups: suspected myofascial pain (n=19) and medication-refractory (n=18). All patients underwent the MPM program, which included instructions for self-massage and stretching techniques. Treatment effectiveness was evaluated using the pain item in the Integrated Palliative Care Outcome Scale (IPOS), with a score of ≤1 indicating improvement.

Results

The overall improvement rate in the patients was 76%, with significant improvements observed in the myofascial (84%; p=0.00017) and refractory (67%; p=0.00038) groups. Of the 28 patients who demonstrated improvements, 25 (89%) achieved this outcome without changing their prescribed headache medications.

Conclusions

The MPM program was effective for patients with chronic headache, including those with medication-refractory headache. Evaluation of the masticatory and pericranial muscles, along with the introduction of a structured self-care program, could prove to be a valuable nonpharmacological strategy for headache management.

## Introduction

Chronic headache is frequently associated with musculoskeletal factors, such as temporomandibular disorders (TMD) and myofascial pain in the masticatory and pericranial muscles. The International Classification of Headache Disorders, 3rd edition (ICHD-3), acknowledges the importance of these factors by recommending physical examination and muscle palpation in its diagnostic criteria [1]. Furthermore, ICHD-3 has introduced “Headache attributed to temporomandibular disorder” (ICHD-3 11.7) as an official diagnostic entity, which is often clinically associated with myofascial pain in the masticatory muscles. However, the effectiveness of structured, nonpharmacological interventions that specifically target these musculoskeletal issues has not been sufficiently validated, particularly for patients with chronic headaches that are refractory to standard drug therapies.

This study aimed to evaluate the clinical effectiveness of a structured myofascial pain management (MPM) self-care program for patients with chronic headaches. In collaboration with the headache specialist clinic in the Department of Neurology at Keio University Hospital, we investigated the program’s impact on two distinct patient groups: those with suspected myofascial involvement and those with medication-refractory headache.

## Materials and methods

Study design and setting

This single-arm, pre-post interventional, observational study was conducted at the Orofacial Pain Clinic within the Department of Dentistry and Oral Surgery at Keio University Hospital, a tertiary care academic medical center. Patient data were collected from July 2020 to July 2023.

Participants

A total of 37 patients with chronic headache were included in the study. All patients were referred from the headache specialist clinic in the Department of Neurology at Keio University Hospital for further evaluation and management of their headache.

Diagnostic procedures and patient categorization

All diagnoses were based on ICHD-3 [1] and the Diagnostic Criteria for Temporomandibular Disorders (DC/TMD) [2]. The diagnostic criteria for “headache attributed to TMD,” a key classification used in this study, are summarized in Table 1. Muscle palpation was performed on the pericranial muscles, as recommended by the ICHD-3, including the frontalis, temporalis, masseter, pterygoid, sternocleidomastoid, splenius, and trapezius muscles. A pressure of 1-2 kgf was applied, and sustained pressure for at least five seconds was used to assess for referred pain. Based on their referral information and clinical presentation, patients were categorized into two groups: a suspected myofascial pain involvement group (n=19) and a medication-refractory group (n=18), which included patients who were either unresponsive to pharmacotherapy or had persistent headache despite some relief.

**Table 1:** Diagnostic criteria for “headache attributed to temporomandibular disorder (TMD) (code 11.7)” based on ICHD-3 ICHD-3: The International Classification of Headache Disorders, 3rd edition [1]

11.7 Headache attributed to temporomandibular disorder (TMD) - diagnostic criteria (ICHD-3)			
A	Any headache fulfilling criterion C		
B	Clinical evidence of a painful pathological process affecting elements of the temporomandibular joint(s), muscles of mastication, and/or associated structures on one or both sides		
C	Evidence of causation demonstrated by at least two of the following:		
	1	The headache has developed in temporal relation to the onset of the temporomandibular disorder, or has led to its discovery	
	2	The headache is aggravated by jaw motion, jaw function (eg, chewing), and/or jaw parafunction (eg, bruxism)	
	3	The headache is provoked on physical examination by temporalis muscle palpation and/or passive movement of the jaw	
D	Not better accounted for by another ICHD-3 diagnosis		

Intervention: the myofascial pain management program

**Figure 1:**
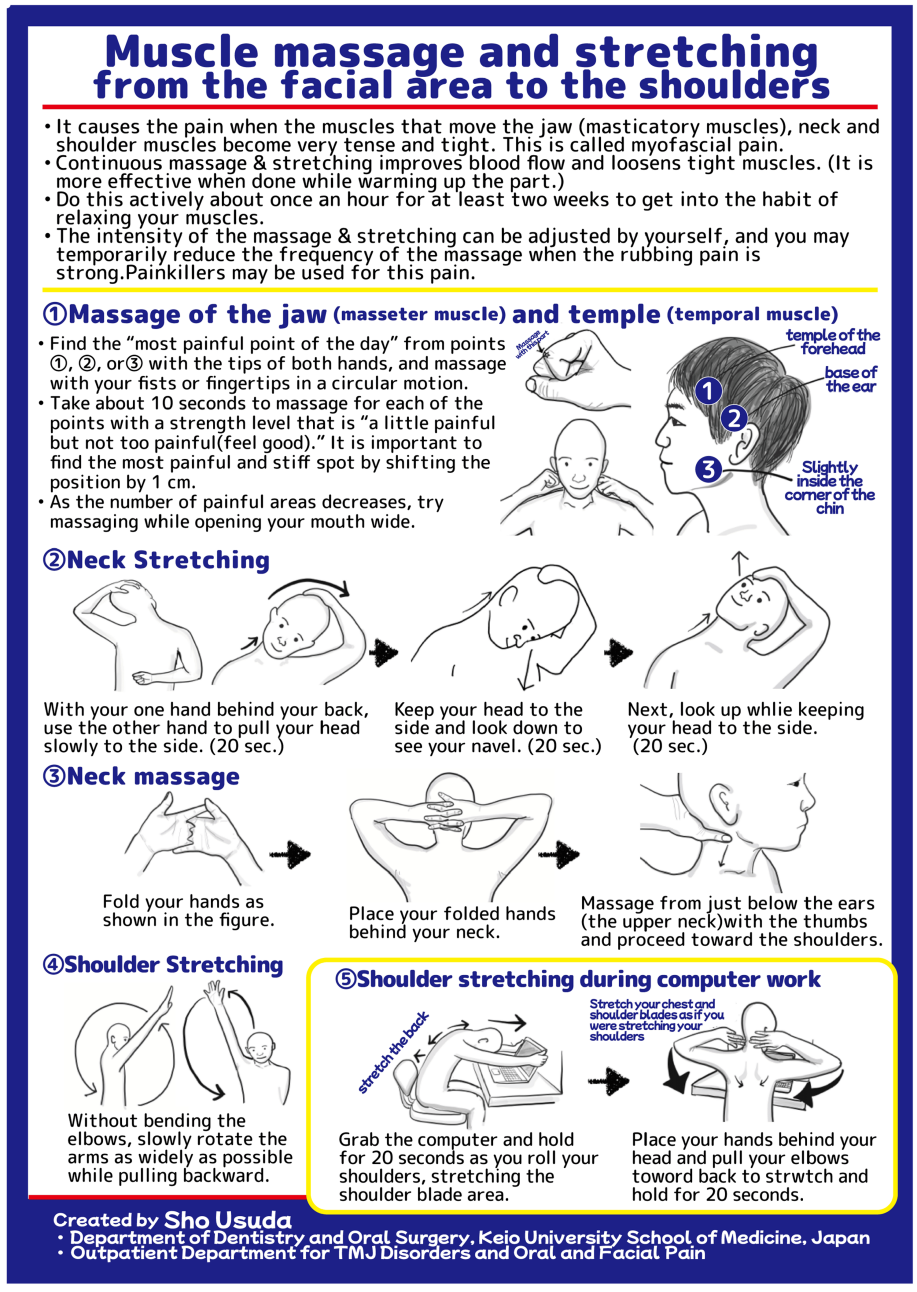
The self-care instruction sheet for the MPM program This illustrated guide provides instructions for self-massage of the jaw muscles (masseter and temporalis), stretching and massage of the neck, and shoulder exercises. Patients were advised to perform these exercises regularly to relieve myofascial tension, which contributes to chronic headaches MPM: myofascial pain management

All patients underwent the structured, multicomponent self-care MPM program. An illustrated instruction sheet summarizing the self-massage and stretching techniques was distributed to all participants (Figure 1). The exercises included in this program were developed based on the findings of previous studies. Accordingly, our program consisted of four key elements: (1) patient education, to facilitate understanding of myofascial pain, including familiar and referred pain reproduced during palpation, with patients directed to educational videos and online resources; (2) self-care instruction, wherein patients received training in self-massage and stretching techniques using real-time feedback, printed instruction sheets (Figure 1), and assistive tools; (3) maintenance of pharmacotherapy, wherein medications prescribed by neurologists were continued without modification to isolate the effects of the MPM program; and (4) follow-up and re-evaluation, wherein improvements in myofascial pain and symptoms were assessed at subsequent visits and self-care instructions were adjusted accordingly.

Outcome measures

The treatment effectiveness was evaluated using the pain item from the healthcare professional version of the Integrated Palliative Care Outcome Scale (IPOS) [3-5]. The IPOS pain item rates pain severity on a 5-point scale ranging from 0 (“not at all”) to 4 (“overwhelmingly”), based on the clinician’s assessment over the past three days. Clinical improvement was defined as a post-intervention IPOS score of ≤1. Assessments were conducted at the initial visit (baseline) and the final follow-up appointment. The IPOS was used under Open Access terms, and no additional permission was required.

Statistical analysis

All statistical analyses were performed with EZR ver 1.61 (Jichi Medical University, Tochigi, Japan), which is a graphical user interface for R (The R Foundation for Statistical Computing, Vienna, Austria). The statistical significance was set at p<0.05. Nonparametric tests were employed due to the non-normal distribution of the IPOS scores, as determined by the Shapiro-Wilk test (p<0.00001), and the unequal variances between groups, confirmed by Levene’s test (p=0.0049). The Wilcoxon signed-rank test was used for pre-post comparisons of IPOS scores within each group. Fisher’s exact test was used to compare the improvement rates between groups, while the Mann-Whitney U test was conducted to examine the changes in IPOS scores (ΔIPOS). Interaction analysis was not performed because the data did not meet the statistical assumptions required for analysis.

Ethical considerations

This study was conducted in accordance with the principles of the Declaration of Helsinki. The study protocol was reviewed and approved by the Institutional Review Board of Keio University School of Medicine (approval number: 20180033). Following a thorough explanation of the study, verbal informed consent was obtained from all participants and documented in their respective medical records.

## Results

Participant characteristics and referral details

**Figure 2:**
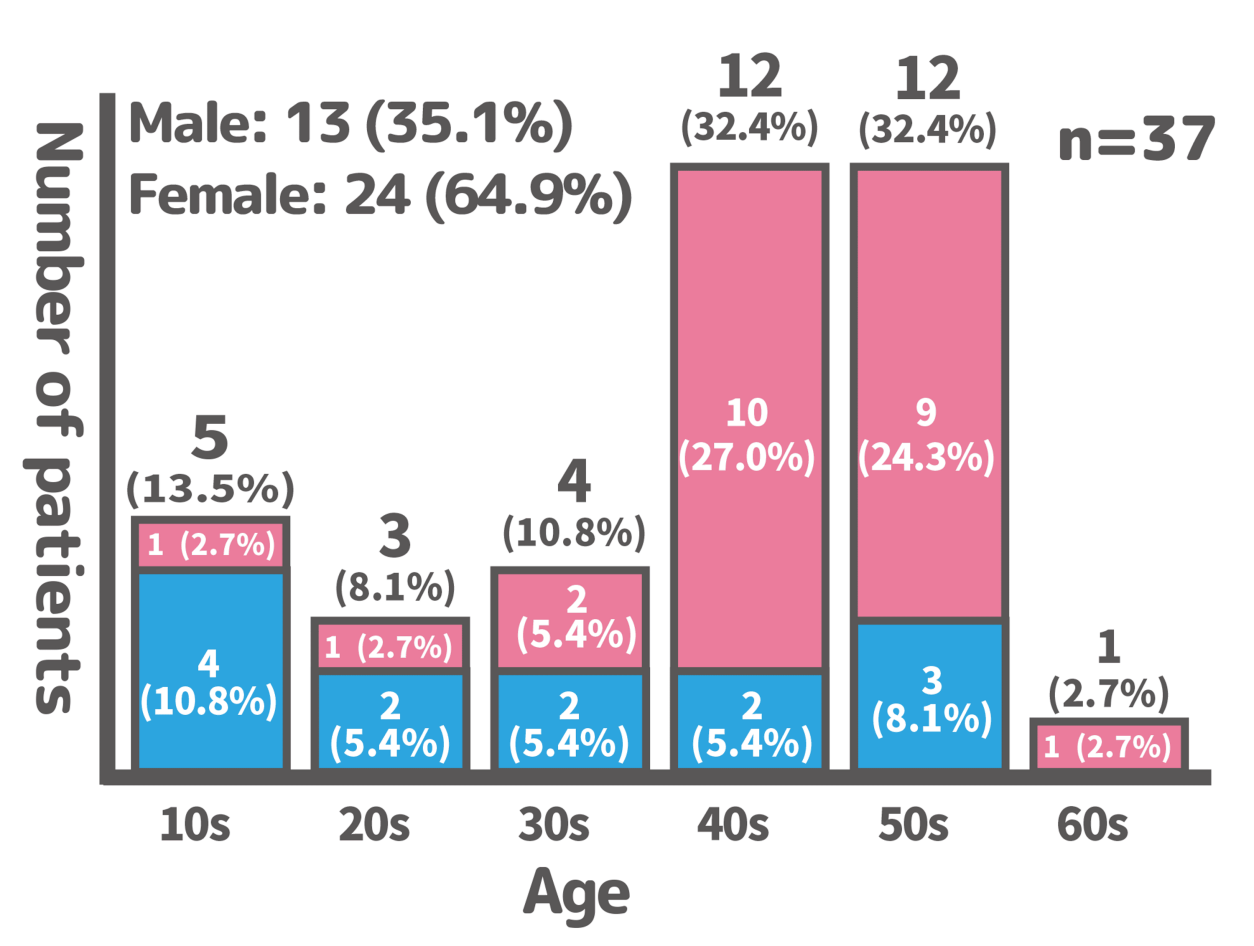
Age and sex distribution of the patients in the study The bar chart illustrates the number of male and female patients in each age group, with females in their 40s and 50s being the most prevalent demographic

A total of 37 patients (13 males, 24 females) were included in this study, resulting in a male-to-female ratio of approximately 1:2. Women in their 40s and 50s represented the most common demographic group (Figure 2).

**Figure 3:**
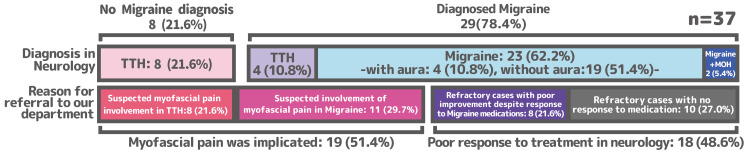
Neurological diagnoses and reasons for referral to our department The chart summarizes the diagnoses established in the neurology clinic - tension-type headache (TTH) (code 2), migraine (code 1), or both - and the reasons for referral to our department, categorized as suspected myofascial pain involvement or medication-refractory headache. MOH (code 8.2): medication-overuse headache

The neurological diagnoses included tension-type headaches (TTH; n=8), migraine (n=23), comorbid TTH and migraine (n=4), and medication-overuse headache (MOH; n=2). Approximately half of the participants (n=19) were referred due to suspected myofascial pain involvement. The remaining patients (n=18) were referred as medication-refractory cases, either due to a lack of response to treatment or persistent headache despite some medication efficacy (Figure 3). A wide range of baseline medications was prescribed by the neurologists. Acute therapies included various triptans (sumatriptan, n=6; naratriptan, n=5; eletriptan, n=4; zolmitriptan, n=1), NSAIDs (loxoprofen, n=4; ibuprofen, n=1), lasmiditan (n=1), and acetaminophen (n=3). Prophylactic agents included calcium channel blockers (lomerizine, n=6), antiepileptics (valproate sodium, n=3; extended-release valproate, n=2), antidepressants (amitriptyline, n=2; duloxetine, n=1), beta-blockers (propranolol, n=2), muscle relaxants (eperisone, n=6; tizanidine, n=1), and CGRP monoclonal antibodies (galcanezumab or erenumab, n=5). Four patients were on no medication at the time of referral.

Myofascial findings and TMD-related diagnoses

**Figure 4:**
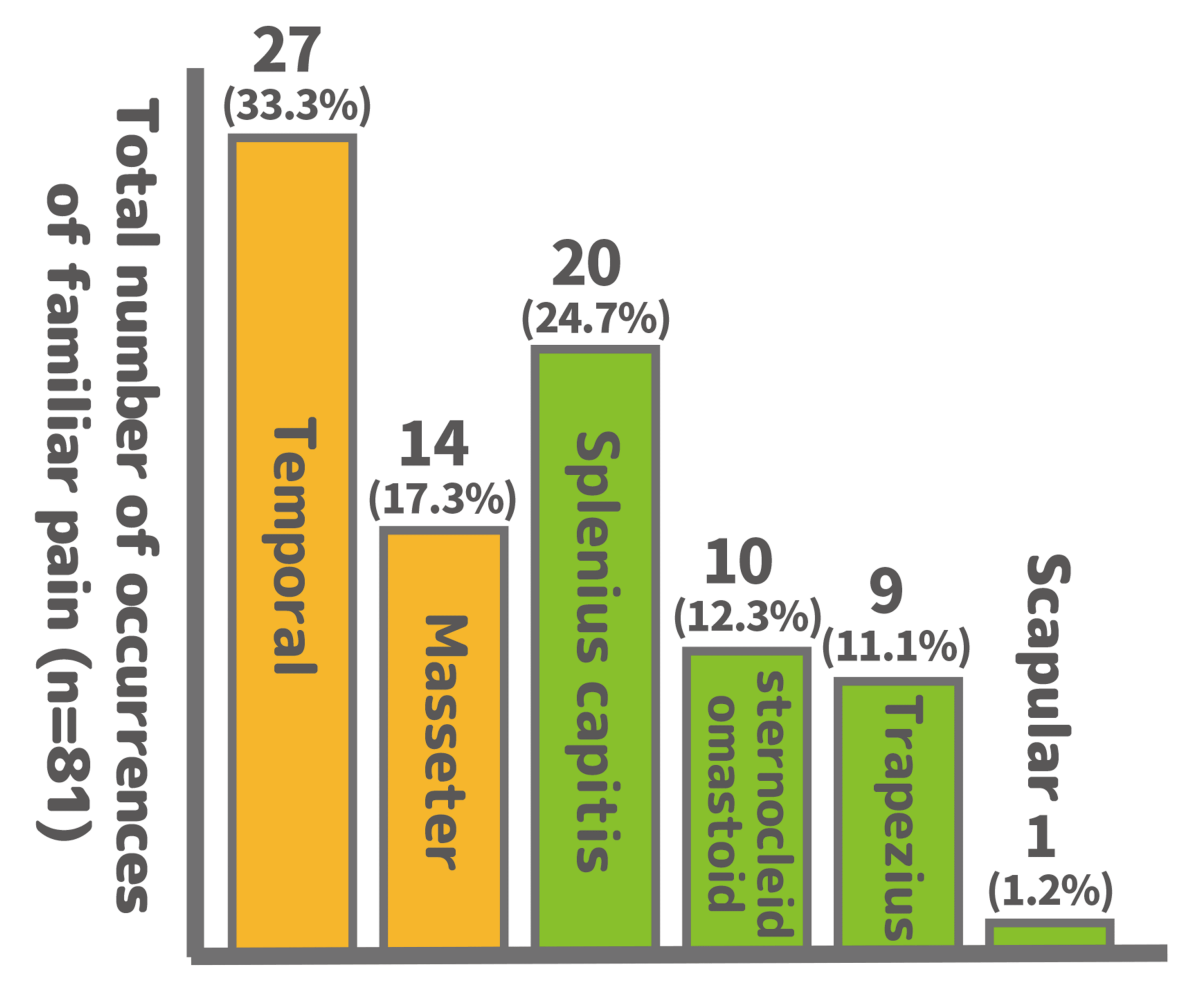
Distribution of muscle sites eliciting familiar pain The vertical axis represents the total number of occurrences in which each muscle elicited familiar pain during palpation (n=81), not the number of unique patients. Percentages are calculated based on the total occurrences: 27 (33.3%), 14 (17.3%), 20 (24.7%), 10 (12.3%), 9 (11.1%), and 1 (1.2%). Only muscle sites that elicited familiar pain are included in the total count. The temporalis and splenius capitis muscles were the most frequently involved ones

Myofascial pain in the pericranial muscles was identified in all 37 patients, including those in the medication-refractory group, in which myofascial pain involvement was not initially suspected. Through standardized muscle palpation, the muscles most frequently identified as sources of familiar pain included the temporalis, splenius capitis, and masseter (Figure 4).

**Figure 5:**
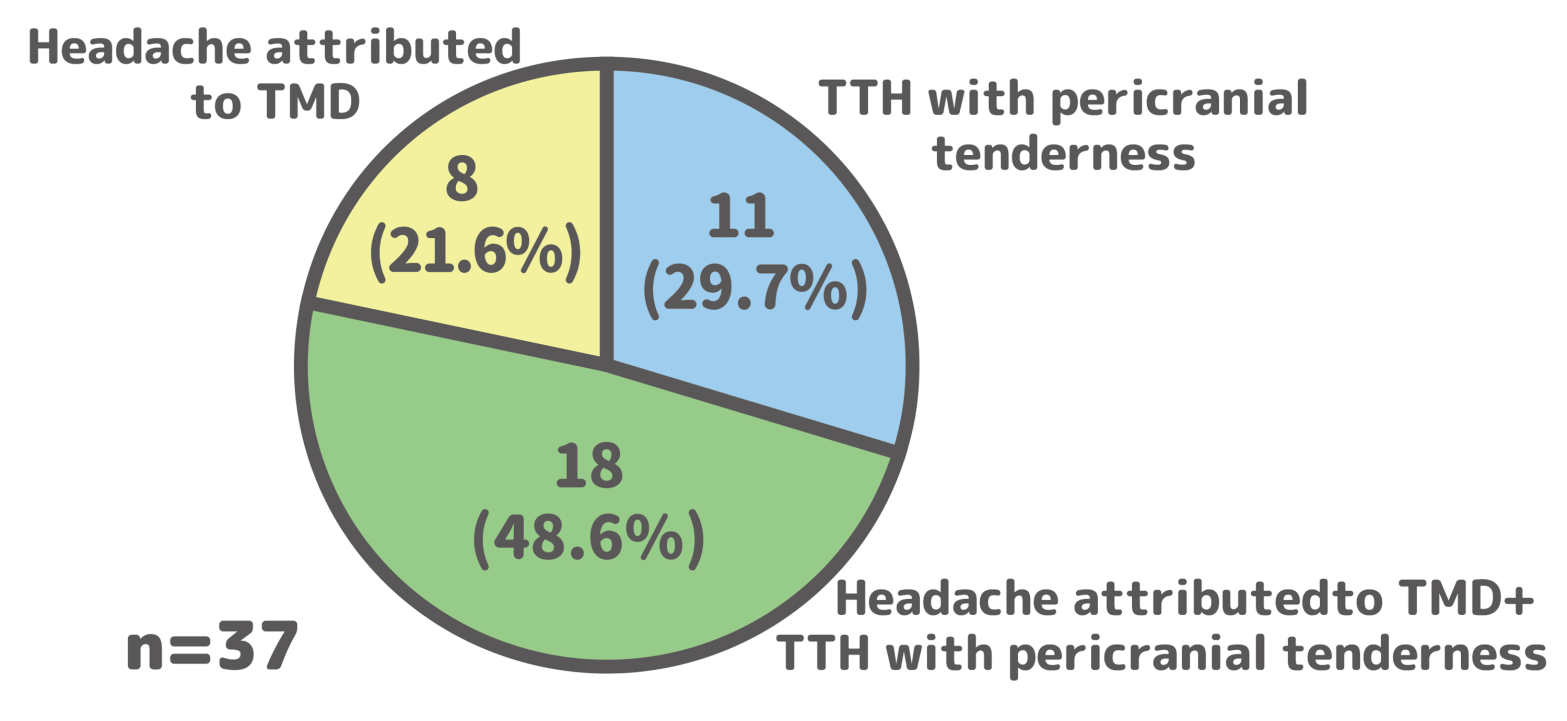
Diagnostic classification based on muscle palpation Pie chart indicating the proportion of patients who met the diagnostic criteria for “headache attributed to temporomandibular disorder (TMD) (code 11.7),” “tension-type headache with pericranial tenderness (TTH) (code 2.4),” or both, based on standardized muscle palpation TTH: tension-type headache

Of the 71 causative muscle sites identified, 41 (58%) were masticatory muscles (temporalis and masseter). Consequently, 26 of the 37 patients (70%) met the diagnostic criteria for “headache attributed to TMD” (Table 1, Figure 5).

These diagnostic classifications were obtained in addition to the initial neurological diagnoses presented in Figure 3 and reflect supplementary evaluations based on muscle palpation performed in our department. The final diagnostic profile for each patient includes the combined findings from both neurological diagnosis (Figure 3) and muscle palpation-based classification (Figure 5).

Treatment effectiveness of the MPM program

**Figure 6:**
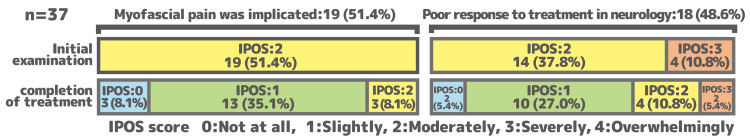
Changes in IPOS pain scores before and after the intervention in both the suspected myofascial pain group (n=19) and the medication-refractory group (n=18) IPOS pain scores were evaluated by healthcare professionals, as described in the Methods section. Values are shown as the number and percentage of patients in each score category. A Wilcoxon signed-rank test confirmed a statistically significant reduction in scores in the myofascial pain group (p=0.00017) and the medication-refractory group (p=0.00038). Between-group comparisons showed no significant differences in improvement rates (Fisher’s exact test, p=0.29) or ΔIPOS (Mann-Whitney U test, p=0.586). Statistical significance was set at p<0.05 IPOS: Integrated Palliative Care Outcome Scale

The overall improvement rate, defined as an IPOS score of 1 post-intervention, was 76% (28 out of 37 patients). High improvement rates were observed in the suspected myofascial pain (84%; 16/19 patients) and medication-refractory (67%; 12/18 patients) groups (Figure 6). The Wilcoxon signed-rank test confirmed a statistically significant reduction in IPOS scores in the myofascial (p=0.00017) and refractory (p=0.00038) groups. No statistically significant differences in improvement rates (Fisher’s exact test, p=0.29) or ΔIPOS (Mann-Whitney U test, p=0.586) were observed between the two groups (Figure 6).

Impact of disease duration on outcomes

**Figure 7:**
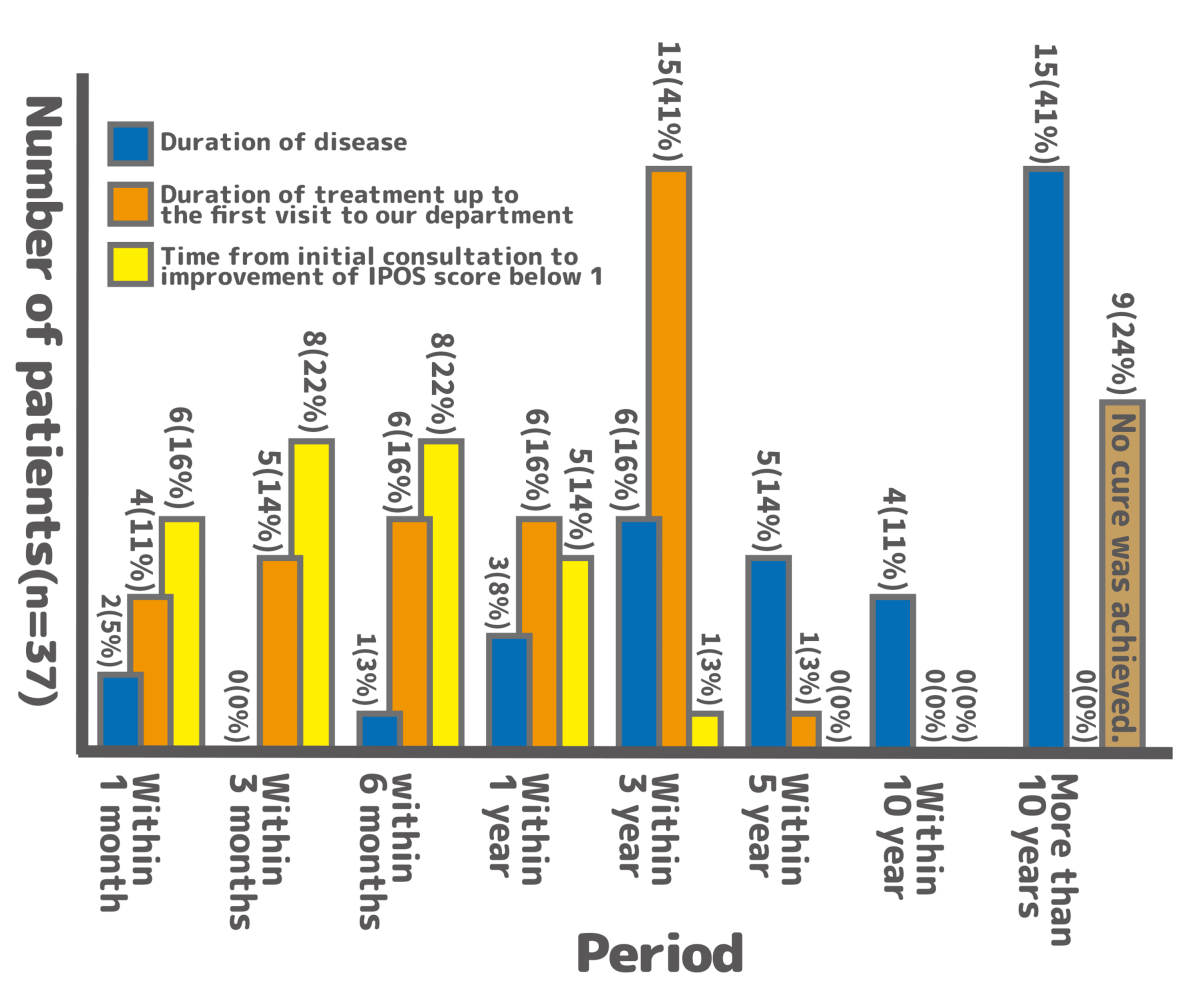
Duration of disease, duration of treatment before the first visit to our department, and time from the initial consultation to improvement of the IPOS score to ≤1 The bar chart uses different colors to represent each of the three time periods. The vertical axis indicates the number of patients (n=37), and the values above each bar show the number and percentage of patients in each category. Among the 30 patients with a disease duration of >1 year, 17 (57%) achieved improvement within a year of the intervention. Among the 24 patients with a duration exceeding three years, 13 (54%) showed improvement within the same timeframe. No statistically significant association was observed between disease duration and the likelihood of improvement (p>0.05). IPOS pain scores were evaluated by healthcare professionals, as described in the Methods section IPOS: Integrated Palliative Care Outcome Scale

The MPM program was effective even in patients with a long history of headaches. Among the 30 patients with a disease duration of >1 year, 17 (57%) achieved improvement within a year of the intervention. Similarly, among 24 patients with a duration exceeding three years, 13 (54%) showed improvement within the same timeframe (Figure 7). The duration of the disease did not have a statistically significant impact on the likelihood of improvement.

Additional observations on treatment outcomes

Of the 28 patients who showed improvement, 25 (89%) did not make any changes to their pre-existing medication regimens prescribed by the neurology department. Among the nine patients who did not report any improvement, four experienced a reduction in their myofascial pain but not in their headaches, while the remaining five did not show any improvement, which was associated with poor adherence to the self-care program despite repeated instruction.

## Discussion

This study revealed two main findings. First, the structured self-care MPM program effectively reduced headache symptoms in patients referred from a neurology clinic, including those with medication-refractory conditions. Second, a substantial proportion of these patients with chronic headache (70%) met the diagnostic criteria for “Headache attributed to TMD” following a standardized muscle palpation examination, a result that aligns with previous research indicating a substantial overlap between headache and TMD [6]. These results indicate that a targeted, nonpharmacological intervention addressing underlying musculoskeletal factors is an effective approach for a broad range of patients with chronic headaches.

Our findings reinforce the clinical importance of the ICHD-3 recommendation for physical examination, as failure to perform muscle palpation may lead to overlooking a treatable factor contributing to the patient’s headache condition. Thus, it is important to assess the masticatory and pericranial muscles during the routine evaluation of chronic headaches. One of the most significant findings of the present study was the effectiveness of the MPM program in the medication-refractory group, which predominantly consisted of patients with migraine.

Therapeutic exercise has been shown to produce analgesic effects, regardless of the specific type of exercise. Further, it exerts widespread hypoalgesic effects beyond the area of application [7,8]. Greater analgesic effects have been observed in specific regions where the intervention was directly applied [9,10]. Therefore, the program used in the current study emphasized targeted approaches to the masticatory, cervical, and shoulder muscles. Moreover, home-based exercise programs have been reported to be as effective as supervised or facility-based interventions [11], supporting the design of this program as a simple and reproducible method. Furthermore, a Cochrane review of interventions for chronic musculoskeletal pain reported that exercise adherence is more strongly influenced by individualized instruction, educational materials, and regular follow-up than by the specific type of exercise performed [12].

The mechanism underlying this effect may be explained by the modulation of central sensitization in the trigeminal nervous system [13-16]. It has been proposed that persistent nociceptive input from myofascial tissues, such as the masticatory muscles, can lead to a state of central hyperexcitability [15]. Preclinical evidence supports this concept; for example, an animal model by Toyama et al. demonstrated that sensitization in the territory of the second trigeminal branch (V2) could enhance neuronal responses and migraine-like behavior related to the first branch (V1) [17]. Given that migraine is primarily a V1-mediated condition and masticatory muscle pain involves the second and third branches (V2/V3), the findings of the present study are consistent with the hypothesis that addressing the peripheral source of pain through the MPM program can attenuate this central sensitization, thereby reducing the frequency or severity of migraine. This neurophysiological rationale is further supported by studies highlighting the role of interbranch trigeminal sensitization in the pathophysiology of chronic migraine [13].

The clinical utility of the MPM program as a nonpharmacological intervention is highlighted by the finding that 89% of patients who demonstrated improvements did not alter their pre-existing medication regimens prescribed by the neurology department. This suggests that the program can exert a therapeutic effect independent of pharmacotherapy. In contrast, among the nine patients who did not improve, five (56%) demonstrated poor adherence to the self-care protocol despite repeated instructions, highlighting that patient adherence is a critical determinant of treatment outcomes in self-management programs. Successful self-management of chronic pain relies not only on performing exercises but also on the patient’s understanding of their condition and the rationale for the treatment, which fosters motivation [18]. Furthermore, interventions that use technology, such as apps and reminders, are effective in enhancing adherence [19]. The structure of our MPM program, which combines education, real-time feedback, and the provision of supportive tools, is designed to foster this crucial element of patient adherence.

This study has several notable strengths. First, its design reflects a real-world clinical setting, based on a close collaboration between a headache specialist clinic in a neurology department and an orofacial pain clinic. This interdisciplinary approach allowed for the systematic evaluation of a patient population that accurately represents a common clinical challenge. Second, the inclusion of a distinct cohort of medication-refractory patients enhanced the clinical relevance of our findings, as this group represented a significant therapeutic challenge. Finally, the use of a structured and protocol-driven intervention, rather than a generalized self-care recommendation, adds to the study’s methodological rigor and provides a clear model for potential clinical implementation.

However, the study has some limitations that should be acknowledged. First, it was conducted with a relatively small sample size (n=37), which may limit the generalizability of our findings to a broader population of patients with chronic headaches. Second, the single-arm, pre-post interventional design without a non-intervention control group made it difficult to definitively exclude the potential influence of the natural history, placebo effects, or regression to the mean on the observed outcomes. Third, the implementation of the self-care program was not quantitatively measured; we relied on patient self-reports without objectively assessing the frequency, duration, or quality of the exercises performed. Finally, our primary outcome measure was limited to a single pain score, and we did not include a comprehensive assessment of other important domains, such as quality of life, functional disability, or psychological well-being.

Nonetheless, this study demonstrates that focusing on myofascial contributors through a structured self-care program can offer a promising avenue for managing chronic headaches. The effectiveness of this approach in patients with difficult-to-treat conditions underscores its clinical value. Moving forward, the key step will be to address the limitations of this study through more robust research designs, such as large-scale randomized-controlled trials with active control groups and comprehensive, long-term assessments. With stronger evidence, the widespread social implementation of this program, supported by public awareness campaigns and accessible tools, such as mobile apps, could offer a valuable preventive and adjunctive strategy in the public health approach to managing chronic headaches.

## Conclusions

A structured self-care program targeting myofascial pain significantly reduced symptoms in a cohort of patients with chronic headaches, including those refractory to medication. The high prevalence of “Headache attributed to TMD” (70%) in this population underscores the clinical importance of this often overlooked musculoskeletal contributor. Therefore, the systematic assessment of masticatory and pericranial muscles, followed by the implementation of a targeted self-care program, represents a valuable and effective strategy for the broader management of patients with chronic headaches.

## Appendices

Appendix A

**Table 2:** IPOS pain item (Healthcare Professional Version)* ^*^Reproduced from [3] under the Open Access CC BY-NC license IPOS: Integrated Palliative Care Outcome Scale

Pain
Over the past three days, has the patient been affected by pain?
Scoring: 0 = not at all, 1 = slightly, 2 = moderately, 3 = severely, 4 = overwhelmingly

## References

[1] (2018). Headache Classification Committee of the International Headache Society (IHS) The International Classification of Headache Disorders, 3rd edition. Cephalalgia.

[2] Ohrbach R, Greene CS, Peck CC (2023). Diagnostic criteria for temporomandibular disorders: Assessment instruments, Version 15. J Oral Facial Pain Headache.

[3] Murtagh FE, Ramsenthaler C, Firth A (2019). A brief, patient- and proxy-reported outcome measure in advanced illness: Validity, reliability and responsiveness of the Integrated Palliative care Outcome Scale (IPOS). Palliat Med.

[4] Moore ZR, Pham NL, Shah JL (2019). Risk of unplanned hospital encounters in patients treated with radiotherapy for head and neck squamous cell carcinoma. J Pain Symptom Manage.

[5] Grochowicka M, Grochowicz K, Drożdżal M (2025). Integrated Palliative Care Outcomes Scale (IPOS): a review of the literature and studies on its use in patients with different conditions. Palliat Med Pract.

[6] Tchivileva IE, Ohrbach R, Fillingim RB, Lin FC, Lim PF, Arbes SJ Jr, Slade GD (2021). Clinical, psychological, and sensory characteristics associated with headache attributed to temporomandibular disorder in people with chronic myogenous temporomandibular disorder and primary headaches. J Headache Pain.

[7] Taylor NF, Dodd KJ, Shields N, Bruder A (2007). Therapeutic exercise in physiotherapy practice is beneficial: a summary of systematic reviews 2002-2005. Aust J Physiother.

[8] Vaegter HB, Handberg G, Graven-Nielsen T (2014). Similarities between exercise-induced hypoalgesia and conditioned pain modulation in humans. Pain.

[9] Naugle KM, Fillingim RB, Riley JL 3rd (2012). A meta-analytic review of the hypoalgesic effects of exercise. J Pain.

[10] Lannersten L, Kosek E (2010). Dysfunction of endogenous pain inhibition during exercise with painful muscles in patients with shoulder myalgia and fibromyalgia. Pain.

[11] Mannion AF, Muntener M, Taimela S, Dvorak J (1999). A randomized clinical trial of three active therapies for chronic low back pain. Spine.

[12] Jordan JL, Holden MA, Mason EE, Foster NE (2010). Interventions to improve adherence to exercise for chronic musculoskeletal pain in adults. Cochrane Database Syst Rev.

[13] Noseda R, Burstein R (2013). Migraine pathophysiology: anatomy of the trigeminovascular pathway and associated neurological symptoms, CSD, sensitization and modulation of pain. Pain.

[14] Ferrillo M, Giudice A, Marotta N (2022). Pain management and rehabilitation for central sensitization in temporomandibular disorders: a comprehensive review. Int J Mol Sci.

[15] Sato H, Saisu H, Muraoka W, Nakagawa T, Svensson P, Wajima K (2012). Lack of temporal summation but distinct aftersensations to thermal stimulation in patients with combined tension-type headache and myofascial temporomandibular disorder. J Orofac Pain.

[16] Merrill RL (2007). Central mechanisms of orofacial pain. Dent Clin North Am.

[17] Toyama M, Kudo C, Mukai C (2017). Trigeminal nervous system sensitization by infraorbital nerve injury enhances responses in a migraine model. Cephalalgia.

[18] Lorig KR, Holman H (2003). Self-management education: history, definition, outcomes, and mechanisms. Ann Behav Med.

[19] Nieuwlaat R, Wilczynski N, Navarro T (2014). Interventions for enhancing medication adherence. Cochrane Database Syst Rev.

